# Is India on a path to reduce the tobacco industry’s influence in tobacco control? Insights from the Global Tobacco Industry Interference Index (2019–2023)

**DOI:** 10.3389/fpubh.2024.1358834

**Published:** 2024-09-19

**Authors:** Sonu Goel, Diksha Walia, Upendra Bhojani, Nandita Bhatnagar, Mrinalima Chopra

**Affiliations:** ^1^Postgraduate Institute of Medical Education and Research, Chandigarh, India; ^2^Faculty of Education and Health Sciences, School of Medicine, University of Limerick, Limerick, Ireland; ^3^Faculty of Human and Health Sciences, Swansea University, Swansea, United Kingdom; ^4^Faculty of India Alliance (DBT/Wellcome Trust), Lead, Cluster on Chronic Health Conditions & Public Policies, Institute of Public Health, Bangalore, India; ^5^Master of Public Health, Panjab University, Chandigarh, India

**Keywords:** tobacco industry, WHO FCTC article 5.3, global tobacco industry interference, India, policy

## Abstract

**Introduction:**

The tobacco industry (TI), driven by profit motives, consistently conceals health risks through deceptive strategies, notably in countries like India. These tactics create vulnerabilities that hinder effective tobacco control measures and enable the TI to exploit legal gaps. Understanding these TI strategies is essential for policymakers to take appropriate preventive and corrective measures in order to limit tobacco industry interference (TII) in policy-making. The study aims at understanding the trend of TII in India between 2019 and 2023.

**Methodology:**

The secondary data from the Global Tobacco Industry Interference report, consisting of seven major domains of the TII index, viz. policy participation, corporate social responsibility (CSR) activities, industry benefits, unnecessary interaction, transparency, conflict of interest, and preventive measures, were retrieved. A composite score was obtained after adding scores of different domains, for each year.

**Results:**

The findings of the study demonstrated an initial improvement in India’s implementation of WHO FCTC Article 5.3, as evidenced by a decreasing score between 2019 and 2021. However, this trend halted in 2023, with data showing a slight increase in the score. When compared with other Asian countries, India shows marginal improvement in score than Cambodia, China, Indonesia, Lao PDR, Malaysia, Myanmar, Nepal, Philippines, Thailand, and South Korea. Some of the countries in the region, including India, Pakistan, Bangladesh, Sri Lanka, Myanmar, Brunei, China, and Vietnam experienced a decline in TII.

**Discussion:**

There has been a rise in CSR activities, forms of unnecessary interactions of TII with policymakers, and participation in policy development; however, improvements are observed in providing benefits to the TI, conflict of interest, and preventive measures. In order to fortify the regulatory framework, it is imperative to create awareness among stakeholders on conflict of interest, denormalize corporate social responsibility (CSR) initiatives by the TI, provision of a watchdog for TII in the country and “whole of government” approach in implementation of FCTC Article 5.3.

## Introduction

The consumption of tobacco products results in over 8 million fatalities annually, including 1.2 million deaths from exposure to second-hand smoke (1). Low- and middle-income countries, accounting for the majority (over 80%) of the world’s 1.3 billion tobacco users, experience the heaviest impact of tobacco-related diseases and deaths. This burden is exacerbated by the fact that households often allocate essential funds meant for necessities such as food, shelter, and children’s education, etc. toward tobacco consumption, leading to increased poverty levels (1). India faces a dual burden in the realm of tobacco consumption, encompassing both smoking and smokeless tobacco making tobacco control, a high priority in the country (2). According to the findings of the Global Adult Tobacco Survey (GATS), conducted in 2016–17, 28.6% of the adult population (42.4% men and 14.2% women) consumes tobacco in various forms (3). National Family Health Survey (NFHS-5) conducted between 2019 and 2021 had similar findings, suggesting that 38% of men consume tobacco, including 28.8% in urban and 42.7% in rural areas, while 8.9% of women consume tobacco including 5.4% in urban and 10.5% in rural areas (4). In addition to the negative effects smoking has on health, a WHO study confirmed the huge economic cost of tobacco-related illnesses and early deaths costs India 1% of its GDP (5). Moreover, the excise taxes on tobacco products yield a loss to the Indian economy of Rs. 816 for every Rs. 100 collected (5).

To maintain its profits and boost sales, the tobacco industry (TI) has always attempted to conceal the negative effects of tobacco consumption on health from the general public (6). Cigarette and smokeless tobacco companies invest billions annually in marketing their products (7, 8). In India, the tobacco products market is anticipated to generate $13,370 million in revenue in 2024, with an annual growth rate of 4.41% from 2024 to 2029 (9). The industry employs approximately 7.25 million people (10) and exported $923.80 million worth of tobacco products in 2021–2022 (11). The TI deploys deceptive strategies exploiting key areas of economic activity, marketing/promotional action, and political activity, and through its manipulative behavior in low- and middle-income countries, including India (12). For instance, Godfrey Philips provided support to flood-affected vendors in Srinagar in the year 2014, creating a deceptive image of being a socially responsible brand (12). Other countries, a fundamental and unresolvable clash exists between the TI’s priorities and public health policies in India (13). The TI has been a significant barrier, undermining the nation’s efforts to implement tobacco control laws. For instance, the TI lobbied for watering and delaying the decision to implement 85% pictorial health warnings (PHWs) in India (14). In another instance, the TI used various strategies to persuade lawmakers and front groups to postpone and divert their attention from the proposed Cigarettes and Other Tobacco Products (Prohibition of Advertisement and Regulation of Trade and Commerce, Production, Supply, and Distribution) Act, COTPA (Amendment) Bill, under the assumption that the amended act would have a detrimental effect on the bidi industry and farmers in the certain states (15). TI in India spends so-called CSR money on primary education, sanitation initiatives, and health promotion initiatives (16–18). In accordance with various government programs at the federal and state levels, the TI also supports agriculture, solid waste management, women’s empowerment, health and sanitation programs, and overall development (16). The initial initiatives by the MoHFW to outlaw ENDS under the Drugs and Cosmetics Act were thwarted by legal challenges and state orders (19). The multinational ENDS giants also provided funding to local ENDS importers to fight the ordinance in court before the country-level ordinance, namely the Prohibition of Electronic Cigarettes Act, 2019 (PECA) was enforced in India (20).

According to Section 135 of the Indian Companies Act, 2013, all companies, including private and limited ones, must spend 2% of their Profit After Tax (PAT) on CSR if they meet any of these criteria: a net worth of Rs. 500 crores or more, a turnover of Rs. 1,000 crores or more, or a net profit of Rs. 5 crores or more. The 2% CSR expenditure is calculated based on the average PAT of the last three financial years (21). FCTC recommends banning TI CSR activities to de-normalize and regulate their so-called “socially responsible” actions (22). During the COVID-19 outbreak, several tobacco companies cumulatively committed approximately US$36.7 million in donations to various government funds, including the “Prime Minister’s Citizen Assistance and Relief in Emergency Situations Fund” (PM CARES Fund) of the Government of India and the Chief Minister’s Relief Fund of different State Governments in the country (23).

The Government of India has ratified the WHO FCTC (21) that outlines important strategies for lowering demand and raising the supply of tobacco, in 2004 (24). Article 5.3 of FCTC aims at safeguarding the policy-making process from the commercial and vested interest of the TI. It seeks to overcome obstacles that hinder the effective implementation of the convention by addressing the issue of TI’s political activities (25). In addition to this, India has undertaken several initiatives, including a lack of specific COTPA enforcement (26), 85% PHW (27), ENDS (Electronic Nicotine Delivery Systems) ban (28), tobacco-free educational institutions guidelines (29), and implementation of the world’s largest national tobacco quitline (30).

On 24 June 2019, the Indian Ministry of Health and Family Welfare issued a letter, signed by Additional Secretary Sanjeeva Kumar, to all state governments in India that emphasized India’s commitment under the WHO FCTC Article 5.3 (31). It cautioned against any collaboration with the Foundation for Smoke-Free World (FSFW), funded by Philip Morris International (PMI), in the interest of public health (31). FSFW and PMI advocate for alternative devices such as e-cigarettes under the guise of promoting a “smoke-free but not vape-free” world (31). The letter also referenced the WHO FCTC Secretariat’s statement, characterizing FSFW as a blatant attempt to undermine the FCTC by interfering in public policy, aimed at undermining treaty implementation (31). These stances reflect the political will to prioritize public health over industry interests. However, the country faces a number of challenges in limiting the influence of the TI, including a lack of specific provisions to deal with industry interference under COTPA 2003, insufficient efforts to implement Article 5.3 of WHO FCTC, influence over policymakers, lack of awareness among policymakers within and outside the health sector, lack of public support, sophisticated marketing strategies adopted by TI, absence of political will, complex legal processes, and international trade benefits (India is the second largest exporter of tobacco after Brazil) (32). These vulnerabilities prevent tobacco control measures from being implemented and monitored effectively and also provide the business room to take advantage of legal weaknesses and work around restrictions. An understanding of the trend of tobacco industry interference (TII) in India will help policymakers and implementors in determining the areas that need additional support in safeguarding public health from the overall detrimental effects of tobacco. The current study uses the Global Tobacco Industry Interference Index to investigate the trend of TII in India between 2019 and 2023.

### What is already known on this topic

The tobacco industry (TI) is known to conceal the harmful effects of tobacco use from the public and use tactics to influence public policies related to tobacco for commercial gains. An extensive tool namely Global Tobacco Industry Interference Index exists which assesses the level of tobacco industry influence by measuring the Implementation of Article 5.3.

### What is unknown on this topic

How exactly (using specific tactics) TI interferes in tobacco control over time in India and what has been government response over time. Understanding and analysing TI strategies along with government response is essential for policy makers to take appropriate preventive and corrective measures in order to limit TII in policy making.

### Policy implications

The findings will help policymakers improve their approaches and bolster their efforts to prevent/minimise TII to safeguard public health.

## Methods

The current study utilizes a comprehensive global dataset to assess TII in India and across Asian countries. The data come from the Global Tobacco Industry Interference Index, an annually updated resource that has expanded its coverage over time.

### Data source

The secondary data originated from the four rounds of the Global Tobacco Industry Interference Index; the first index in 2019 encompassed 33 countries, followed by the second index in 2020 covering 57 countries, and subsequently the third index in 2021 covering 80 countries and fourth index in 2023 evaluating 90 countries. The country’s ranking was executed using the identical questionnaire and scoring approach as the ASEAN Index, devised by the Southeast Asia Tobacco Control Alliance (33). The Global Center for Good Governance in Tobacco Control (GGTC), situated at the School of Global Studies, at Thammasat University, acts as the leading center with support from Stopping Tobacco Organizations and Products (STOP), Thai Health Promotion Foundation, and the Bill and Melinda Gates Foundation (facilitates the assessment of countries and the formulation of the index) (34), which assists in assessing countries and formulating the index. The index is based upon a publicly accessible dataset concerning TII within countries, as well as the responses of their respective governments and civil society organizations about the domains of the index to such interference (34).

### Study variables

The Global Tobacco Index (or Global Tobacco Industry Interference Index) rates a nation according to how governments address the industry, incorporating preventative measures (34). The seven major domains of the index are Participation in Policy Development, Tobacco-Related CSR Activities, Benefits to Tobacco Industry, Forms of Unnecessary Interaction, Transparency, Conflict of Interest, and Preventive Measures (34–37).

### Data analysis

A composite score was obtained after adding the scores for various domains of the Global Tobacco Industry Interference Index for each year. A lower score indicates a reduced level of overall interference, which is considered beneficial for the country’s public health efforts (34).

### Ethics considerations

The study relies on publicly available secondary data, eliminating the need for informed consent. Data privacy and confidentiality are ensured by utilizing aggregated information. Since no direct interaction with human participants took place, the study does not involve potential harm to individuals.

## Results

A total of 33, 57, 80, and 90 nations were represented in the first (2019), second (2020), third (2021), and fourth (2023) rounds of the Global Tobacco Index report respectively. The TI’s involvement in policy development in India has been on the rise, increasing from a score of 6 in 2019 to a consistent 7 in 2020, 2021, and 2023. The industry’s involvement takes the form of advisory groups in public health policy and exhibits an increase from three points in 2019 to five points in 2020, 2021, and 2023. The score of tobacco-related CSR initiatives as per Recommendation 6.2 (The government agencies/officials endorse, form partnerships with/participate in TI CSR activities) also increased from 4 in 2019 and 2020 to 5 in 2021 and 2023. The government’s support for the TI, as outlined in Recommendation 7.3 (The government gives privileges, incentives, exemptions, or benefits to the TI), has consistently held at a score of five for three consecutive years and has experienced a decrease to four points in the year 2023. The level of interaction between the industry and the government has shown a fluctuating pattern, beginning at 12 in 2019, dropping to 9 in 2020, rebounding to 11 in 2021, and further increasing to 14 in 2023. Transparency in government interactions with the industry has decreased, falling from 9 in 2019 to 10 in both 2020 and 2021, and further declining to 9 in 2023. Meanwhile, the score for conflict of interest has consistently dropped from 12 in 2019 to 10 in 2020 and has been a consistent 9 in 2021, and 2023. Notably, preventive measures demonstrated significant improvement over the 5-year span, plummeting from a rating of 21 in 2019 to 10 in 2021 and remaining the same in 2023.

Throughout the 5-year period, India’s overall score consistently reflects an improving trend, beginning at 69 in 2019, declining to 61 in 2020, further decreasing to 57 in 2021, and ultimately settling at 58 in 2023. The reduction of scores in the preventative measures was done through increased transparency in its dealings with the TI (Recommendation 5.1), implementing a code of conduct for public officials when dealing with the TI (Recommendation 4.2), by asking the TI to disclose information on tobacco production and manufacturing and other activities including lobbying, philanthropy, and political contributions periodically (Recommendation 5.2) (Table 1).

**Table 1:** Trend analysis using the global tobacco industry interference index from 2019 to 2023 in India.

Parameters of assessment	Maximum possible score	Year of assessment			
		2019	2020	2021	2023
Participation in policy development	20	6	7	7	7
The government accepts, supports, or endorses offers for assistance by or in collaboration with the tobacco industry in implementing tobacco control policies (Recommendation 3.1)	5	2	1	1	1
The government accepts, supports, or endorses legislation drafted by/in collaboration with the tobacco industry (Recommendation 3.4)	5	0	0	0	0
The government allows the tobacco industry to sit in multi-sectoral committee/advisory group that sets public health policy (Recommendation 4.8)	5	3	5	5	5
The government allows representatives from the tobacco industry (including State-owned) in the delegation to the COP or subsidiary bodies or accepts their sponsorship for delegates. (Recommendations 4.9 and 8.3)	5	1	1	1	1
Tobacco related CSR activities	5	4	4	5	5
The government receives contributions from the tobacco industry (including so-called CSR contributions) (Recommendation 6.4) The government agencies/officials endorse, form partnerships with/participate in tobacco industry CSR activities (Recommendation 6.2)	5	4	4	5	5
Benefits to the tobacco industry	10	5	5	5	4
The government accommodates requests from the industry for longer implementation time or postponement of tobacco control law (Recommendation 7.1)	5	0	0	0	0
The government gives privileges, incentives, exemptions, or benefits to the tobacco industry (Recommendation 7.3)	5	5	5	5	4
Forms of unnecessary interaction	15	12	9	11	14
Top-level government officials meet with/ foster relations with the tobacco companies such as attending social functions and events sponsored or organized by the tobacco companies (Recommendation 2.1)	5	3	2	3	5
The government accepts assistance/offers of assistance from the tobacco industry on enforcement (Recommendations 3.1 and 4.3)	5	5	3	4	4
The government accepts, supports, endorses, or enters into partnerships or agreements with the tobacco industry (Recommendation 3.1)	5	4	4	4	5
Transparency	10	9	10	10	9
The government does not publicly disclose meetings/ interactions with the tobacco industry where such interactions are strictly necessary for regulation (Recommendation 2.2)	5	5	5	5	5
The government requires rules for the disclosure or registration of tobacco industry entities, affiliate organizations, and individuals acting on their behalf including lobbyists.	5	4	5	5	4
Conflict of interest	15	12	10	9	9
The government does not have a policy (whether or not written) to prohibit contributions from the tobacco industry or any entity working to further its interests to political parties, candidates, or campaigns or to require full disclosure of such contributions (Recommendation 4.11)	5	4	5	4	4
Retired senior officials work for the tobacco industry (Recommendation 4.4)	5	4	5	5	5
Current government officials and their relatives hold positions in the tobacco business including consultancy positions (Recommendations 4.5, 4.8, and 4.10)	5	4	0	0	0
Preventive measures	25	21	16	10	10
The government has a procedure for disclosing records of the interaction with the tobacco industry and its representatives (Recommendation 5.1)	5	4	4	2	2
The government has formulated, adopted, or implemented a code of conduct for public officials, prescribing the standards they should comply with when dealings with the tobacco industry (Recommendation 4.2)	5	4	4	2	2
The government requires the tobacco industry to periodically submit information on tobacco production, manufacture, market share, marketing expenditures, revenues, and any other activity, including lobbying, philanthropy, and political contributions (Recommendation 5.2)	5	5	2	2	2
The government has a program/system/plan to consistently raise awareness within its departments on policies relating to FCTC Article 5.3 Guidelines (Recommendation 1.1 and 1.2)	5	3	2	2	2
The government has a policy prohibiting the acceptance of all forms of contributions from the tobacco industry (monetary or otherwise) including offers of assistance, policy drafts, or study visit invitations to the government, officials, and their relatives (Recommendation 3.4)	5	5	4	2	2
Total	100	69	61	57	58

**Figure 1:**
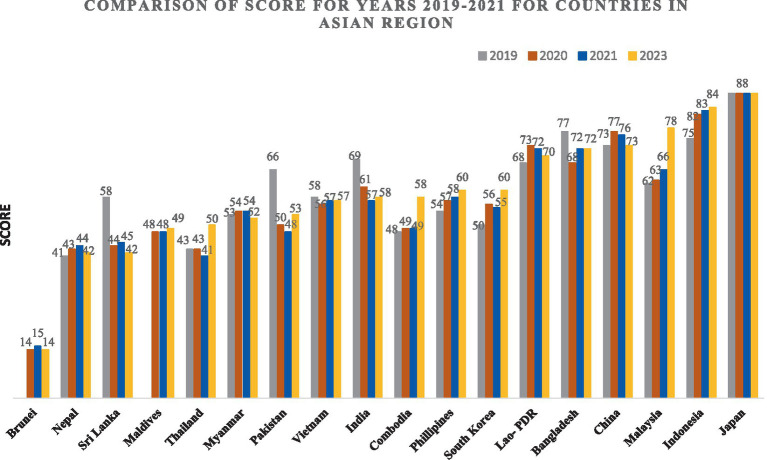
Comparison of global TII scores for 3 years (2019–2021) for countries in the Asian Region.

The implementation of Article 5.3 in the Asian region exhibits an irregular trend, as demonstrated by the scores obtained from the Global Tobacco Industry Interference Index for the years 2019–2023. Several countries, including India, Pakistan, Bangladesh, Sri Lanka, Myanmar, Brunei, China, and Vietnam, have demonstrated improvements in their scores. Conversely, countries such as Cambodia, China, Indonesia, Lao PDR, Malaysia, Nepal, the Philippines, Thailand, and South Korea have seen declines in their performance. The overall trends depict varied efforts in tobacco control across the region, with some countries making progress and others facing challenges (Figure 1).

## Discussion

The current study employs the Global Tobacco Industry Interference Index to evaluate trends in TII in India. The findings of the study demonstrated an initial improvement in India’s implementation of WHO FCTC Article 5.3, as evidenced by a decreasing score between 2019 and 2021. However, this trend halted in 2023, with data showing a slight increase in the score.

### Participation in policy development

Despite being a signatory to the convention for 15 years, India has not been able to establish a national policy for all government officials that effectively prevents interference from the TI (38). Many countries such as Iran, Korea, Nepal, Kenya, the UK, Uganda, and Uruguay have set commendable examples by excluding the TI from policy-making discussions and rejecting any form of support, collaboration, or input from the industry when developing and implementing public health policies (36, 38, 39). In India, though the TI is not part of policy development, indirect lobbying efforts and political favors influence the policy (40). In addition, health is a state subject in India, so many state governments have taken proactive initiatives to control the interference of the TI. For example, the High Court of Karnataka demanded the Tobacco Board of India to withdraw its participation and funding from a TI event, in addition to asking governments to consider a “code of conduct” for dealing with the TI (41). To date, 22 states of India have enacted a protocol for public employees, banning the exchange of favors or any cooperation between a public agency and the tobacco business, hence limiting the interactions between public officials and the TI (42).

### Tobacco-related CSR activities

India’s corporate sector, including cigarette corporations, is mandated to allocate a minimum of 2% of profits to corporate social responsibility (CSR) activities under the Companies Act if the net worth of the company is more than 500 crore or their annual turnover is above 1,000 crores or the net profit is above 500 crores (43). As this rule also applies to some cigarette companies, a challenging situation has emerged because the social welfare initiatives of these companies may indirectly encourage tobacco use. To manage this shortcoming in the Companies Act, both the Cigarettes and Other Tobacco Products Act (2003) and FCTC recommend banning TI CSR activities to de-normalize and regulate their so-called “socially responsible” actions (22). The government has witnessed a gradual rise in the contributions received from the TI, and an increase in collaborations between the government and the industry for CSR initiatives over time (23). In one of the instances Indian tobacco businesses contributed close to US$37 million to government coffers as part of COVID-19 relief initiatives (23), for enhancing their corporate image and generating profits, thereby contravening the provisions under COTPA 2003 and FCTC Article 5.3. These CSR activities conducted by Indian tobacco companies involve the utilization of their corporate trademarks, which are also present on their respective tobacco products. As a case in point, ITC employs the “ITC” trademark across all its tobacco and non-tobacco goods, while “Godfrey Phillips” and “DS Group” employ the same trademark for their entire product range. These instances of CSR activities conducted by Indian tobacco companies, especially the usage of company trademarks, violate not only Section 5(3)(b) of COTPA 2003 but also Article 13 of the WHO Framework Convention on Tobacco Control (WHO FCTC) and the associated implementation guidelines (12, 23). For example, in response to a legal petition, one of the high court of India has categorically outlined that the cigarettes and TI, in the course of their CSR activities, cannot breach the provisions outlined in the COTPA, 2003 (44).

### Benefits to the tobacco industry

WHO Article 5.3 demanded that the countries should refrain from providing advantages or incentives to the TI (36). In the countries, where CSR activities were reportedly intensive such as Malaysia, Pakistan, Tanzania, and Zambia, it was observed that these countries did not impose any tax increases on tobacco (36). In a similar fashion, India was unable to make progress in ceasing the provision of benefits to the TI, as evidenced by a consistent score of five points maintained between 2019 and 2021. The evidence suggests that the Indian government continues to grant privileges, incentives, exemptions, or benefits to the TI (34–36). However, there has been improvement in the period from 2022 to 2023, with the score decreasing to four. The introduction of India’s Goods and Services Tax (GST) in 2017 brought about substantial changes to the system of indirect taxation. All tobacco products became subject to the highest slab (28% GST) with an additional compensation cess for cigarettes and smokeless tobacco products (45). However, for some states, the newly introduced taxes were less as compared to the old VAT regime (45). Moreover, the beedi (hand-rolled tobacco wrapped in specific tendu leaves) industry enjoys the status of a cottage industry and remains out of this tax slab. Furthermore, companies with <20 employees or small tobacco farmers and exporters are also exempted from tax (40). Such exemption should be withdrawn and uniform taxation should be introduced to all tobacco products in line with WHO recommended taxation of 75% on the retail price of tobacco products (46).

### Forms of unnecessary interactions

Unwarranted engagements take place when high-ranking government officials attend social events organized by tobacco companies or when the government embraces offers of assistance or forms partnerships with the TI (34). The TI has gained notoriety for its resourcefulness in maintaining relationships with governments worldwide (47). In 2015, India’s largest cigarette manufacturer provided funding for the 10th Sustainability Summit held in the capital city of New Delhi (47). This tobacco company has a long-standing association with the CII-ITC Center of Excellence for Sustainable Development, which regulates these events with partnerships with various government ministries, including Housing and Urban Poverty Alleviation and Environment, Forests and Climate Change, and GIZ (German Society for International Cooperation, Ltd.) (47). Few prominent figures from the TI often held key positions in these summits, frequently alongside policymakers. A representative from the United Nations Development Program (UNDP) was also listed as a speaker (47). This summit evoked memories of the World Business and Development Awards, an initiative supported by UNDP to recognize private sector entities striving to achieve the Millennium Development Goals (47).

### Transparency

The significance of transparency is emphasized in WHO Article 5.3, which urges governments to establish mechanisms that mandate the TI to provide regular disclosures regarding their activities and practices. However, the government falls short of ensuring such obligations from the TI, as evident from the rise in the overall score from nine in 2019 to 10 in both 2020 and 2021. Additionally, there has been a rise in the score from four in 2019 to five in both 2020 and 2021, followed by a decline to four in 2023 regarding the need for the government to implement rules regarding the disclosure or registration of TI entities, affiliate organizations, and individuals acting on their behalf, including lobbyists (34–37).

### Conflict of interest

The guidelines outlined in Article 5.3 recommend the avoidance of conflicts of interest among government officials and employees, along with the establishment of rules to safeguard public health policies from interference by the TI (34). To prevent industry influence on tobacco control policies and programs, India’s Ministry of Health and Family Welfare adopted a code of conduct in 2020 after 13 states implemented Article 5.3; however, the scope of application is restricted to health ministry officials (48). A former member of the Indian Administrative Service (IAS) who held several high-level positions in the Ministries of Communications, Information Technology, and Home Affairs in India has joined the Godfrey Philips board as an independent director (49). A former Honorable President of India was the chief guest at the Confederation of Indian Industry (CII)-ITC Sustainability Awards event in 2012, which presents a conflict of interest (13). Furthermore, in 2011, the Chairman of one large tobacco company was bestowed with the Padma Bhushan, which is India’s third highest civilian award, thereby highlighting a possible contradiction between their official positions and their associations with the TI (13). Over the course of 5 years, the Indian government has demonstrated progress in addressing conflicts of interest, as reflected in the decrease in scores from 12 in 2019 to 10 in 2020 and further to 9 in 2021 and 2023.

### Preventative measures

The guidelines outlined in Article 5.3 offer a variety of measures that governments can implement to safeguard their tobacco control policies against interference from commercial and vested interests (36). The adoption of a code of conduct for officials dealing with the TI, the implementation of transparency and accountability procedures for interactions, and the prohibition of accepting any kind of contributions—including technical assistance—from the TI are just a few proactive steps that governments can take to protect their officials from exposure to interference (39). Several nations, including the Philippines, the United Kingdom, and Australia, have implemented a code of conduct guiding public official’s interactions with the TI. Following them in July 2020 India has also embraced a similar code of conduct for public officials engaging with the TI (50). This indicator showed the most improvement among all indicators over a 5-year period, reducing from a score of 21 in 2019 to 10 in 2023. However, the national policy, titled “Code of Conduct for Public Officials,” applies only to officials of the Ministry of Health and Family Welfare, limiting its scope (51). This restriction contradicts Article 5.3 guidelines, creating a barrier to effective implementation (13). A broader national policy is needed, aligning with state initiatives, to establish effective multilevel governance for tobacco control (13). Despite the fact that various states have codes of conduct for dealing with public officials across the government; however, they are limited in their implementation and are variable, which are not fully compliant with a recommendation under WHO FCTC Article 5.3 (52).

Despite laudable efforts by India over the last few decades to improve TII scores, there have been increasing rates of cancers. The etiology of cancer is quite complex and is driven by multiple variables including demographic, social, economic, and cultural factors. Effective tobacco control measures usually show their positive impact on cancer incidence only after a decade or more. Therefore, it is not unusual to see enhanced tobacco control measures coexisting with increasing cancer incidence and current prevalence rates. Factors such as age, alcohol consumption, exposure to carcinogens, chronic inflammation, diet, hormonal changes, immunosuppression, infectious agents, obesity, radiation, and sunlight exposure all contribute to the multifaceted nature of cancer risk, highlighting that focusing solely on tobacco control may not be sufficient to reduce overall cancer rates (53). However, accelerated tobacco-control programs, especially in areas where usage is increasing, will be crucial in reducing the rates of tobacco-related cancer mortality (54).

The top tobacco companies operating in India ITC, British American Tobacco’s Indian affiliate, Godfrey Phillips India holds a dominant 90% share of the Indian manufactured cigarette market (55, 56). These figures indicate significant market power and revenue for these companies (55, 56). Historically, Indian tobacco companies spent heavily on marketing and advertising before restrictions, demonstrating substantial financial resources. During the COVID-19 pandemic, tobacco companies made significant donations and CSR contributions, contributing approximately $36.7 million (23). ITC alone committed $13.2 million to the PM CARES Fund and established a $19.8 million contingency fund, reflecting their considerable financial capability (23). Moreover tobacco companies have also continued to influence the government through sponsorship and CSR activities, as highlighted in the India Tobacco Industry Interference Index 2020, indicating their financial capacity to engage in policy-influencing activities.

In the regional context, India’s scores positioned it mid-way, akin to countries such as Pakistan, Vietnam, and the Philippines. Another group of countries, including Thailand, Nepal, Sri Lanka, and Maldives exhibited lower scores (41–50) than India demonstrating a significant improvement in implementation of Article 5.3 of FCTC. Conversely, a cluster of significant economies, including Malaysia, Bangladesh, Lao PDR, China, Indonesia, and Japan, registered higher scores than India (70–88). Notably, India and Sri Lanka made commendable progress, with scores dropping from 69 to 58 and 58 to 42, respectively.

### Limitations

The current study draws patterns from the Global Tobacco Industry Interference Index which only includes data that is readily accessible to the public limiting its scope (36). A major limitation is the reliance on publicly available information. In a country like India, which is vast, multilingual, and has numerous media outlets, as well as many business and government entities operating at various levels and scales, it is challenging to gather and scrutinize all potential evidence of TII present in the public domain. For example, many studies on TII in India have only examined English-language media and have only marginally considered selected regional language media. This limitation is particularly significant in India’s complex landscape. Due to its vast size, linguistic diversity, numerous media outlets, and many business and government entities, comprehensively gathering and analyzing all potential evidence of TII is extremely challenging. Many Indian TII studies have primarily focused on English-language media, with only limited exploration of regional language sources. It is challenging to gather comprehensive data on industry interference, which is a crucial provision of the FCTC rules because of a lack of transparency. Some of the TI’s interference and lobbying activities would have become virtual with the introduction of pandemic-related lockdowns and movement restrictions in many countries, making them less transparent and more difficult to monitor and document (36). Furthermore, the global index provides aggregate scores at the indicator level. In addition, it is limited regarding access to specific TII instances and/or examples of preventive measures scored in order to arrive at indicator-level scores. The lack of such granular-level insights limits us in making more specific recommendations/interpretations.

## Conclusion

It is essential that India should develop a national policy specifically designed to prevent TII, as a standalone policy or embedded in the prevailing national tobacco control legislation (COTPA). This policy should be uniformly applicable and enforceable across all departments/agencies across national government and governments in every state and union territory. Further raising awareness among non-governmental organizations, governmental institutions, development sector partners, and elected leaders about TII and measures to prevent the same is of paramount importance. The establishment of a dedicated watchdog entity to monitor the TI’s activities, particularly its attempts to influence policy agenda-setting and implementation, is crucial, and so are the measures for the protection of whistle-blowers.

In addition to this, it is essential to go beyond the existing code of conduct for public officials and address structural and policy-level conflicts, such as those arising from the mandate of the Tobacco Board of India or investments by Public Sector Undertakings (PSUs) in the TI, bringing policy coherence across different government agencies in the interest of public health. Policymakers should also prioritize efforts to denormalize and prevent the so-called CSR by the TI that currently seems to promote the industry’s social image and access to decision-making space. Instead, such mandatory financial contributions from the industry could be directed by governments toward tobacco control-related activities, while ensuring that their CSR and/or environmental, social, and governance (ESG) activities are not exploited for publicity gains.

Strengthening the mechanisms for implementing and enforcing tobacco control policies is critical to ensure that improved TII scores translate into reduced industry interference in tobacco control and correspondingly stronger and effective tobacco control measures leading to positive health impacts. Increased support for research examining the long-term impact of tobacco control policies is essential to bridge the gap between policy implementation and health outcomes. Finally, bolstering cooperation with other countries and international organizations to share best practices and strategies in combating TII should be a key priority.

## Data Availability

The original contributions presented in the study are included in the article/supplementary material, further inquiries can be directed to the corresponding author.

## References

[1] World Health Organization. Tobacco. (2023) Available at: https://www.who.int/news-room/fact-sheets/detail/tobacco (Accessed June 26, 2023).

[2] Bhatia M Sharma N Saifi S Parashar S Nisha N Srivastava R. Evolution of tobacco control in India: a narrative review of the legislative and regulatory approach. Rev Environ Health. (2022) 39:1–12. doi: 10.1515/reveh-2022-0160 36103211

[3] Round S. Tobacco Survey India. Global adult tobacco Survey GATS 2. (2016-2017) Available at: https://ntcp.mohfw.gov.in/assets/document/surveys-reports-publications/Global-Adult-Tobacco-Survey-Second-Round-India-2016-2017.pdf (Accessed June 26, 2023).

[4] Ministry of Health and Family Welfare, National Family Health Survey (NFHS-5), 2019–21. Available at: https://dhsprogram.com/pubs/pdf/FR375/FR375.pdf (Accessed July 10, 2023).

[5] World Health Organization. India loses 1% of its GDP to diseases and early deaths from tobacco use, finds WHO study. (2023) Available at: https://www.who.int/india/news/detail/09-02-2021-india-loses-1-of-its-gdp-to-diseases-and-early-deaths-from-tobacco-use-finds-who-study (Accessed December 8, 2023).

[6] Tobacco Industry Interference a Global Brief. (2012) Available at: www.who.int (Accessed July 11, 2023).

[7] Trade Commission F. Federal Trade Commission Cigarette Report for 2019. (2024) Available at: https://www.ftc.gov/cigarettedata (Accessed July 3, 2024).

[8] Trade Commission F. Federal Trade Commission Smokeless Tobacco Report for 2019. (2024) Available at: https://www.ftc.gov/smokelesstobaccodata (Accessed July 3, 2024).

[9] Tobacco Products - India | Statista Market Forecast. (2024) Available at: https://www.statista.com/outlook/cmo/tobacco-products/india (Accessed July 3, 2024).

[10] Estimates of Tobacco-dependent Employment in India | Economic and Political Weekly. (2024) Available at: https://www.epw.in/journal/2018/40/notes/estimates-tobacco-dependent-employment.html (Accessed July 27, 2024).

[11] Tobacco Industry. (2024) Available at: https://www.indiantradeportal.in/vs.jsp?lang=0&id=0,31,24100,24123 (Accessed July 4, 2024).

[12] Goel S Kar SS. Report on tobacco industry interference in India-case studies. (2022) Available at: https://www.researchgate.net/publication/362508404 (Accessed July 04, 2023).

[13] Rao NV Bhojani U Shekar P Daddi S. Conflicts of interest in tobacco control in India: an exploratory study. Tob Control. (2016) 25:715–8. doi: 10.1136/tobaccocontrol-2015-052503, PMID: 26612763

[14] Oswal KC Pednekar MS Gupta PC. Tobacco industry interference for pictorial warnings. Indian J Cancer. (2010) 47:101–4. doi: 10.4103/0019-509X.65318 20622423

[15] Final-Report-on-Tobacco-Industry-Interference-in-India-Case-Studies1.

[16] ITC Limited. ITC: Creating Enduring Value Focus: ITC’s World-class Indian brands Board of Directors and Committees 01 Report on Corporate Governance 12 Shareholder Information 28. 2018. Available at: https://www.itcportal.com/about-itc/shareholder-value/annual-reports/itc-annual-report-2018/pdf/ITC-Report-and-Accounts-2018.pdf (Accessed July 17, 2023).

[17] Godfrey Phillips India - Best Tobacco Manufacturer in India. (2023). Available at: https://www.godfreyphillips.co/wp-content/uploads/2018/02/CSR-Report-2016-17.pdf (Accessed August5, 2023).

[18] World Health Organization. Tobacco industry interference with tobacco control. Geneva: World Health Organization (2008).

[19] Exclusive: India Proposes Ban on e-Cigarettes, With Jail Terms for Offenders - Government Documents | Reuters. (2023). Available at: https://www.reuters.com/article/us-india-ecigarettes-exclusive/exclusive-india-proposes-ban-on-e-cigarettes-with-jail-terms-for-offenders-government-documents-idUSKCN1VC1RI (Accessed August 6, 2023).

[20] PM’s Remarks at the UNGA High-Level Meeting on Universal Health Coverage | Prime Minister of India. (2023). Available at: https://www.pmindia.gov.in/en/news_updates/pms-remarks-at-the-unga-high-level-meeting-on-universal-health-coverage/ (Accessed August 6, 2023).

[21] Sarkar R. Corporate Social Responsibility: An Indian Perspective. (2019). Parikalpana: KIIT Journal of Management. 15:141. Available at: 10.23862/KIIT-PARIKALPANA/2019/V15/I1-2/190179 (Accessed August 10, 2024).

[22] World Health Organization. Article 5.3 Resources & Tools | GGTC. (2024). Available at: https://ggtc.world/knowledge/article-5.3-resources-%26-tools (Accessed July 17, 2023).

[23] Yadav A Lal P Sharma R Pandey A Singh RJ. Tobacco industry corporate social responsibility activities amid COVID-19 pandemic in India. Tob Control. (2022) 31:777–80. doi: 10.1136/tobaccocontrol-2020-056419 33853882

[24] WHO Framework Convention on Tobacco Control overview. (2023). Available at: https://fctc.who.int/who-fctc/overview (Accessed July 5, 2023).

[25] Fooks GJ Smith J Lee K Holden C. Controlling corporate influence in health policy making? An assessment of the implementation of article 5.3 of the World Health Organization framework convention on tobacco control. Global Health. (2017) 13:1–20. doi: 10.1186/s12992-017-0234-8 28274267 PMC5343400

[26] COTPA 2003 and Rules Made Thereunder: National Health Mission. (2023). Available at: https://nhm.gov.in/index4.php?lang=1&level=0&linkid=459&lid=692 (Accessed August 22, 2023).

[27] Ministry of Health and Family Welfare 2014. 2014-Amendment-Rules-GSR-727E. (2023). Available at: https://clpr.org.in/wp-content/uploads/2018/10/2014-Amendment-Rules-GSR-727E.pdf (Accessed August 31, 2023).

[28] Tobacco Industry Tactics: Packaging and Labelling. (2023). Available at: https://www (Accessed August 7, 2023).

[29] Ministry of Health and Family Welfare. Tobacco Free Educational Institution. Available at: https://ntcp.mohfw.gov.in/assets/document/TEFI-Guidelines.pdf (Accessed July 10, 2023).

[30] National Tobacco Control Programme. (2023). Available at: https://ntcp.mohfw.gov.in/national_tobacco_quit_line_services (Accessed August 31, 2023).

[31] India Heightens Efforts to Protect Health Policies Against Tobacco Industry Vested Interests | WHO FCTC. (2024). Available at: https://extranet.who.int/fctcapps/fctcapps/fctc/kh/TIInterference/news/india-heightens-efforts-protect-health-policies-against (Accessed July 4, 2024).

[32] Tobacco Industry. (2023). Available at: https://www.indiantradeportal.in/vs.jsp?lang=0&id=0,31,24100,24123 (Accessed July 27, 2024).

[33] Assunta M Dorotheo EU. SEATCA tobacco industry interference index: a tool for measuring implementation of who framework convention on tobacco control article 5.3. Tob Control. (2016) 25:313–8. doi: 10.1136/tobaccocontrol-2014-051934, PMID: 25908597 PMC4853530

[34] Global Tobacco Index. (2023). Available at: Available from: https://globaltobaccoindex.org/about (Accessed August 23, 2024).

[35] Global Tobacco Industry Interference Index (2020). Available at: www.globaltobaccoindex.org (Accessed July 13, 2023).

[36] Global Tobacco Industry Interference Index 2021 Acknowledgements. (2021). Available at: www.globaltobaccoindex.org (Accessed October 04, 2023).

[37] Assunta M Dorotheo U Rosa D Sy D Sandberg E. Global tobacco industry interference index 2023 Acknowledgements additional editorial input: global Center for Good Governance in tobacco control and vital strategies. (2023). Available at: www.globaltobaccoindex.org (Accessed October 10, 2023).

[38] Prevent Tobacco Lobby Interference | Deccan Herald. (2023). Available at: https://www.deccanherald.com/opinion/panorama/prevent-tobacco-lobby-interference-872603.html (Accessed July 13, 2023).

[39] Chunhui W Xuewen J Ren C Jiani S Khow K. Acknowledgements Main country collaborators Bangladesh: PROGGA knowledge for Progress Brazil: tobacco for control Alliance Cambodia: Cambodia movement for Health Canada: action on smoking & health China: consultants. (2023). Available at: www.globaltobaccoindex.org (Accessed October 19, 2023).

[40] Chugh A Bassi S Nazar GP Bhojani U Alexander C Lal P. Tobacco industry interference index: implementation of the World Health Organization’s framework convention on tobacco control article 5.3 in India. Asia Pac J Public Health. (2020) 32:172. doi: 10.1177/1010539520917793 32396402 PMC7612145

[41] Bhojani U Venkataraman V. Public Policies and the Tobacco Industry. (2024). Available at: https://www.researchgate.net/publication/262414140 (Accessed 10 August, 2024).

[42] Public Policies and the Tobacco Industry | Economic and Political Weekly. (2023). Available at: https://www.epw.in/journal/2011/28/commentary/public-policies-and-tobacco-industry.html (Accessed November 30, 2023).

[43] Ministry of Corporate Affairs. Annexure Frequently Asked Questions (FAQs) on Corporate Social Responsibility (CSR). Available at: https://www.mca.gov.in/Ministry/pdf/FAQ_CSR.pdf (Accessed August 08 2024).

[44] Cyril Alexander S. Union of India | Tobacco Control Laws. (2023). Available at: https://www.tobaccocontrollaws.org/litigation/decisions/s-cyril-alexander-v-union-of-india-2 (Accessed November 30, 2023).

[45] John RM Dauchy E Goodchild M. Estimated impact of the GST on tobacco products in India. Tob Control. (2019) 28:506–12. doi: 10.1136/tobaccocontrol-2018-054479, PMID: 30219796

[46] Promoting Taxation on Tobacco Products. (2023). Available at: https://www.who.int/europe/activities/promoting-taxation-on-tobacco-products (Accessed December 20, 2023).

[47] Manipulation by association: tobacco, food and public health in India. (2023). Available at: https://www.internationalhealthpolicies.org/blogs/manipulation-by-association-tobacco-food-and-public-health-in-india/ (Accessed July 14, 2023).

[48] Dental Council of India(DCI). Code of conduct for public officials in compliance to Article 5.3 of WHO FCTC. Available at: https://dciindia.gov.in/Admin/NewsArchives/Website%20L.No.1534.PDF (Accessed November 19, 2023).

[49] Re-Appointment-of-Statutory-Auditor. (2023). Available at: https://www.godfreyphillips.co/public/storage/media/Re-appointment-of-Statutory-Auditor.pdf (Accessed November 16, 2023).

[50] Ministry of Health and Family Welfare. Code of conduct for public officials in compliance to Article 5.3 of WHO FCTC. Available at: https://smokelesstobaccocontrolindia.com/wp-content/uploads/2020/07/Code-of-Conduct-for-Public-Officials-6th-July.pdf (Accessed November 19, 2023).

[51] Ministry of Health and Family Welfare. Code of Conduct for Public Officials 2.cdr.

[52] Bassi S Ralston R Arora M Chugh A Nazar GP Collin J. Understanding the dynamics of notification and implementation of article 5.3 across India’s states and union territories. Tob Control. (2022) 31:s18–25. doi: 10.1136/tobaccocontrol-2021-057119 35140171 PMC9125360

[53] Risk Factors for Cancer - NCI. (2024). Available at: https://www.cancer.gov/about-cancer/causes-prevention/risk (Accessed July 7, 2024).

[54] Anand P Kunnumakara AB Sundaram C Harikumar KB Tharakan ST Lai OS. Cancer is a preventable disease that requires major lifestyle changes. Pharm Res. (2008) 25:2097. doi: 10.1007/s11095-008-9661-9 18626751 PMC2515569

[55] Godfrey P. (2024). Ups market share on higher volumes amid tepid demand|Company News - Business Standard. Available at: https://www.business-standard.com/article/companies/godfrey-philips-ups-market-share-on-higher-volumes-amid-tepid-demand-120021101392_1.html (Accessed July, 04 2023).

[56] The Cigarettes-Crunch During the Long Lockdown is Likely to Force People to Cut Down – But Those Who Continue Will End Up Paying More | Business Insider India. (2024) Available at: https://www.businessinsider.in/business/news/cigarettes-crunch-during-the-long-lockdown-will-force-people-to-cut-down-but-smokers-will-end-up-paying-more/articleshow/75426177.cms (Accessed July 4, 2024).

